# Essential Oil from *Cryptomeria japonica* Induces Apoptosis in Human Oral Epidermoid Carcinoma Cells via Mitochondrial Stress and Activation of Caspases

**DOI:** 10.3390/molecules17043890

**Published:** 2012-03-30

**Authors:** Jeong-Dan Cha, Ji-Young Kim

**Affiliations:** 1 Department of Dental Hygiene, College of Natural Sciences, Dongeui University, Busan 614-714, Korea; 2 Department of Dental Hygiene, Ulsan College, San 160-1, Hwajeong-Dong, Dong-Gu, Ulsan 682-715, Korea

**Keywords:** *Cryptomeria japonica*, essential oil, apoptosis, mitochondrial stress, caspases

## Abstract

*Cryptomeria japonica* D. Don (*C. japonica*) has been used in traditional medicines from Asia for a variety of indications, including liver ailments, and an antitussive, and for its antiulcer activities. We examined the cell viability and apoptosis of KB cells treated with *C. japonica* essential oil at several concentrations for 12 h by MTT assay, Hoechst-33258 dye staining, DNA fragmentation, flow cytometry (cell cycle), and Western blotting for mitochondria stress, activation of caspases, and poly (ADP-ribose) polymerase. The essential oil induced the apoptosis of KB cells in a dose-dependent manner, which was verified by DNA fragmentation, appearance of apoptotic bodies, and the sub-G1 ratio. The essential oil also induced rapid and transient caspase-3 activity and cleavage of PARP of the KB cells. Treating the cells with the oil also caused changes in the mitochondrial level of the Bcl-2 family proteins such as Bcl-2 and Bax, thereby inducing the release of cytochrome *c *into the cytosol. The essential oil of *C. japonica* may have potential as a cancer chemopreventive and therapeutic agent.

## 1. Introduction

Apoptosis is an important phenomenon in cytotoxicity induced by anticancer drugs. The execution of apoptosis, or programmed cell death, is associated with characteristic morphological and biochemical changes mediated by a series of gene regulation and cell-signaling pathways [1,2,3]. The mitochondrial apoptotic pathway plays a pivotal role in the apoptotic cell death [1,4]. The release of cytochrome *c* from mitochrondria in response to proapoptotic signals has been suggested as an initiating event in the apoptotic process [4,5]. Cytochrome *c* released from mitochondria is associated with apoptosis protease activating factor (Apaf-1) and pro-caspase-9, triggering the activation of caspase-3 and resulting in cell death [5,6,7,8]. The inhibition of apoptosis, a universal and efficient cellular suicide pathway, is a hallmark characteristic of cancer [9,10].

Sugi, *Cryptomeria japonica* D. Don (*Taxodiaceae*), is an important forest tree because of its excellent characteristics, including rapid growth, straight bole, ready regeneration, and soft wood with a pleasant color and scent. In addition, various constituents of *C. japonica* have been employed as herbal medicines. The leaves include bioactive compounds, since cedar leaves contain essential oils and flavones, among other compounds [11,12,13]. The essential oil from the leaves of *C. japonica* inhibits the formation of gastric mucosal lesions in rats, and several terpenes from this essential oil have shown antiulcer activities [13,14]. In a previous study, we isolated the essential oil from *C. japonica*, and identified its chemical composition using gas chromatography (GC)/mass spectrometry (MS) analyses. The results showed that the essential oil of *C. japonica* contains 68 compounds, representing 95.82% of the total oil content, with α-pinene (6.07%), sabinene (8.86%), terpinen-4-ol (9.77%), α-terpineol (6.13%), elemol (11.17%) and 10(15)-cadinen-4-ol (7.16%), comprising the main portion of the oil. In addition, the essential oil exhibits potent antimicrobial activity against many types of facultative and obligate anaerobic bacteria [15]. Furthermore, the oil was demonstrated to possess excellent antitermite and larvicidal effects against mosquito larvae [16,17].

This study investigated the possible mechanism(s) of the apoptosis mediated by the essential oil of *C. japonica*. The results demonstrate for the first time that the essential oil induces apoptosis in the KB cells through a mitochondria- and caspase-dependent mechanism.

## 2. Results

### 2.1. Effect of Essential oil on KB Cell Proliferation

**Figure 1:**
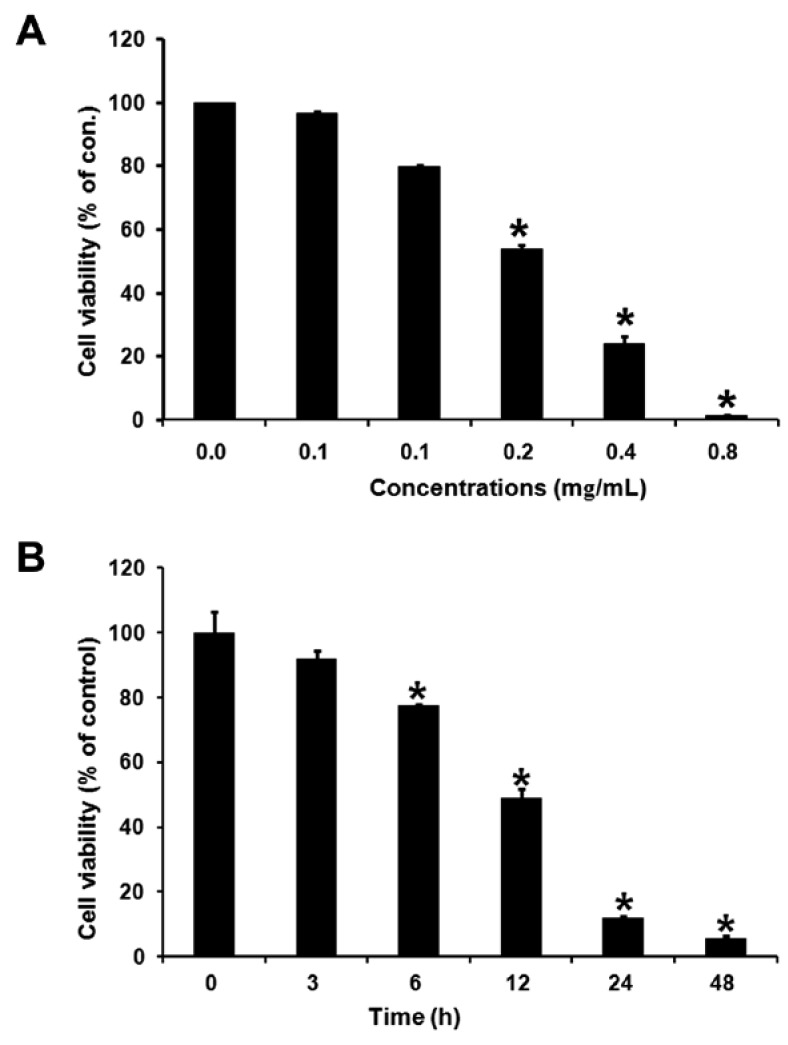
Effect of essential oil from *Cryptomeria japonica *on cell proliferation in KB cells. KB cells were plated into 24 well plates and treated several times with different concentrations (**A**) of the essential oil (**B**). Cell proliferation was determined by the MTT assay and is expressed as percentage of the absorbance value obtained without essential oil. The results are expressed as the mean ± S.E. from three different experiments with triplicate cultures. * *p* < 0.05 compared with control.

KB cells were treated several times with the essential oil at various concentrations, and cell viability was determined using the MTT assay. As shown in Figure 1, essential oil inhibited the growth of KB cells in a dose- and time-dependent manner. Cell viability was obviously inhibited by a 12-h treatment of 0.2 mg/mL essential oil (*p *< 0.05).

### 2.2. Effect of Essential Oil on Cell Cycle Phase Distribution in KB Cells

**Figure 2:**
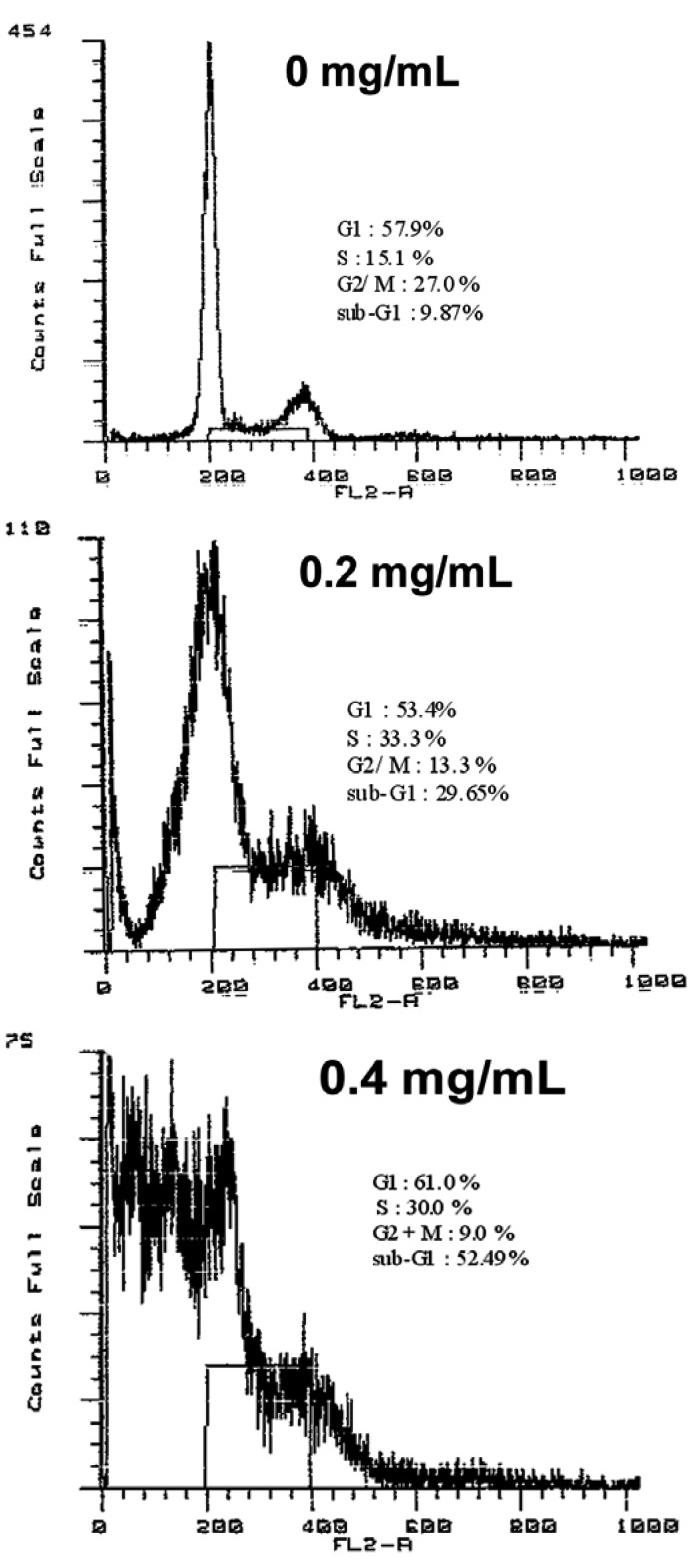
Effect of the essential oil of *Cryptomeria japonica *on cell cycle phase distribution in KB cells. KB cells were treated for 12 h with 0.2 and 0.4 mg/mL of the essential oil. Following the treatment, cells were collected for three different experiments examining apoptosis induction. The percentages of apoptotic cells were determined by propidium iodide staining followed by flow cytometric analysis. Data are representative of at least three independent experiments.

The redistribution of cell cycle phases was analyzed after treatment with various concentrations of the essential oil for 12 h. The proportion of cells in the G2/M-phase was decreased in essential oil-treated cells when compared with control. The number of cells in G0/G1 and S-phase was increased in essential oil-treated cells at concentrations of 0.2 and 0.4 mg/mL when compared with control. Cells with sub-G1 DNA content, a hallmark of apoptosis, were seen in the essential oil-treated group following 12 h exposure at concentrations of 0.2 and 0.4 mg/mL (Figure 2).

### 2.3. Effect of Essential Oil on Determination of Morphological Changes in KB Cells

**Figure 3:**
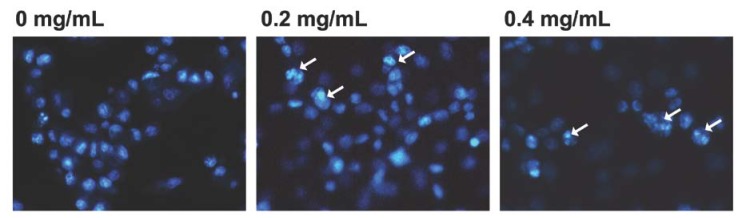
Effect of the essential oil of *Cryptomeria japonica *on determination of morphological changes in KB cells. KB cells were treated with 0.2 and 0.4 mg/mL of the essential oil for 12 h. Following the treatment, cells were collected for three kinds of experiments for apoptosis induction. The morphologic change was assessed by fluorescence microscopy after staining with Hoechst 33258. The apoptotic cells are indicated with arrows. Normal nuclear morphology is observed in untreated cells. In contrast, small, fragmented, and condensed nuclei with typical apoptotic morphology were observed in treated cells.

Nucleic acid staining with Hoechst 33258 revealed typical apoptotic nuclei, which exhibited highly fluorescent condensed chromatin in cells treated with the essential oil (Figure 3). The morphological changes and cell death of KB cells were significantly increased in a dose-dependent manner at essential oil concentrations of 0.2 and 0.4 mg/mL. Most cells were detached from the dishes, and cell rounding and shrinking occurred at the same concentrations of the essential oil (Figure 3).

### 2.4. Effect of Essential Oil on DNA Fragmentation in KB Cells

**Figure 4:**
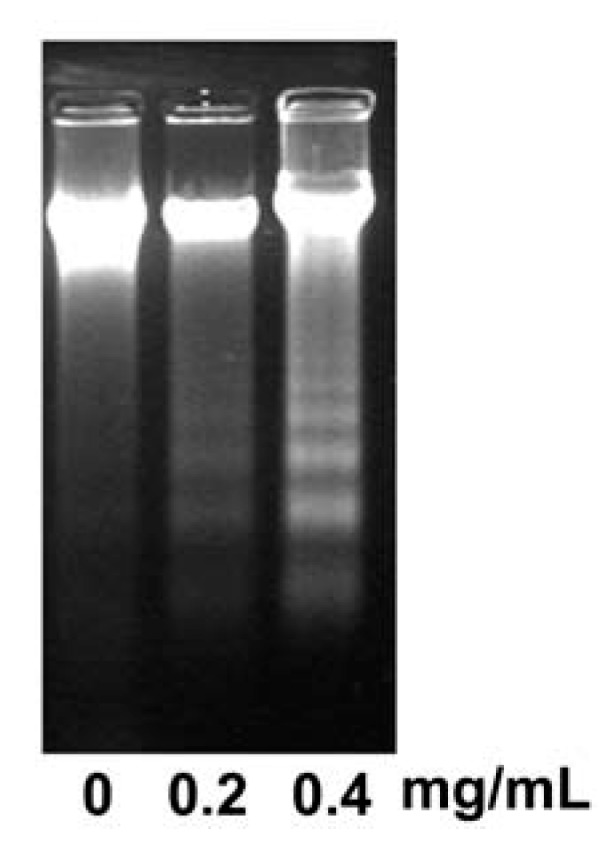
Effect of the essential oil of *Cryptomeria japonica *on DNA fragmentation in KB cells. KB cells were treated with 0.2 and 0.4 mg/mL of the essential oil for 12 h. Following the treatment, cells were collected for three kinds of experiments assessing apoptosis induction. For DNA fragmentation analysis, intracellular DNA was isolated and analyzed by 2.0% agarose gel electrophoresis.

To determine whether the essential oil could induce apoptosis in KB cells, we assessed DNA fragmentation, which is a biochemical hallmark for apoptosis. Essential oil induced endonucleolytic DNA cleavage in a dose-dependent manner (Figure 4). The efficient induction of apoptosis was observed at essential oil concentrations of 0.2 and 0.4 mg/mL in KB cells treated for 12 h.

### 2.5. Essential Oil-Mediated Apoptosis of KB Cells Involves a Caspase-Dependent Mechanism

**Figure 5:**
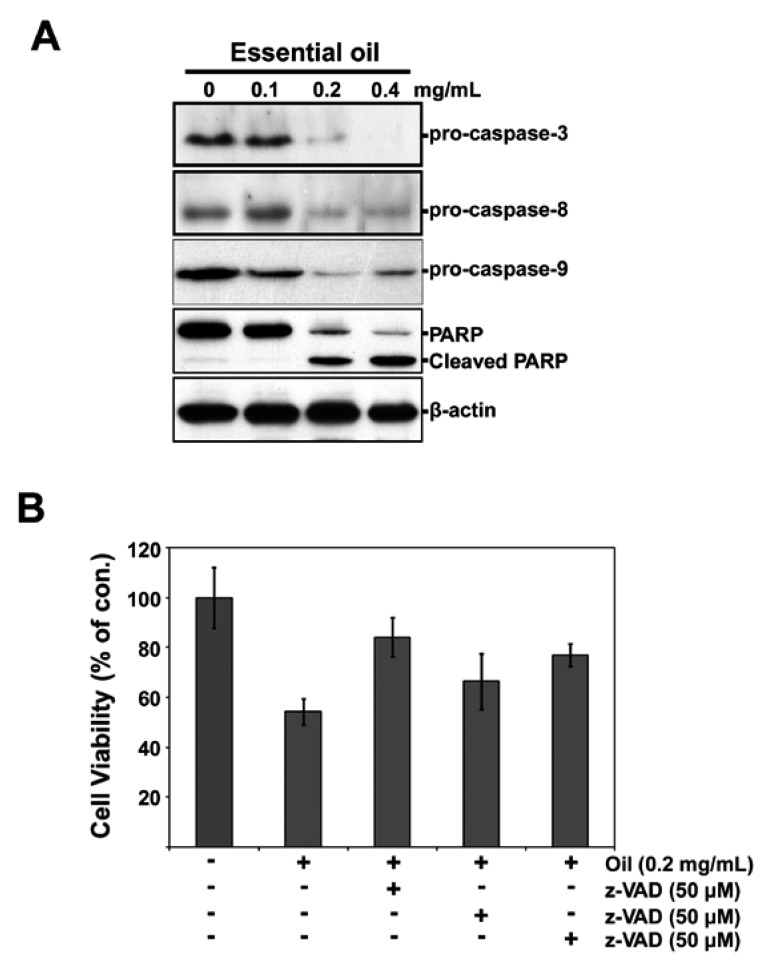
Essential oil-mediated apoptosis of KB cells involves a caspase-dependent mechanism. (**A**) KB cells were exposed to the indicated concentrations of the essential oil for 12 h, and the expression of several procaspases was determined by Western blot analysis; (**B**) The cells were also pretreated with 50 μM of caspase inhibitors z-VAD-fmk, z-IETD-fmk, or z-LEHD-fmk for 1 h before exposing them to 0.2 mg/mL of the essential oil and incubating for an additional12 h. MTT-reducing activity was then determined.

Caspase activities were measured by Western blot analyses because the activation of caspases such as caspase-3, -8, and -9 is one of the most common processes occurring in apoptotic signaling events [19]. As shown in Figure 5A, a dose-dependent activation of caspase-8 was observed after treating KB cells with the essential oil. In particular, approximately 50% and 70% degradation of procaspase-8 and -9 were observed when the cells were exposed to 0.2 and 0.4 mg/mL of the essential oil for 12 h, respectively. In contrast, the oil-mediated degradation of procaspase-3 was more apparent than that of procaspase-8 and -9. KB cells were then treated with 0.2 mg/mL of the essential oil for 12 h in the presence or absence of 50 μM of z-VAD-fmk, z-IETD-fmk or z-LEHD-fmk to further confirm that caspase activation plays a critical role in the oil-mediated apoptotic process. These inhibitors significantly inhibited the oil-induced cytotoxicity in the cells (Figure 5B). In particular, the inhibitory effect of z-VAD-fmk or z-LEHD-fmk was higher than z-IETD-fmk. This suggests that the caspase-dependent pathway is closely related to the oil-induced cell death of KB cells. To further confirm the apoptosis induced by the essential oil, we investigated the cleavage of poly (ADP-ribose polymerase poly (PARP) in KB-treated cells. Treatment of KB cells with 0.2 and 0.4 mg/mL essential oil caused a proteolytic cleavage of PARP, with accumulation of the characteristic 85-kDa fragments and a concomitant disappearance of the full-length 116-kDa protein (Figure 5).

### 2.6. Mitochondrial Stress is an Important Event in the Essential Oil-Mediated Apoptosis of KB Cells

**Figure 6:**
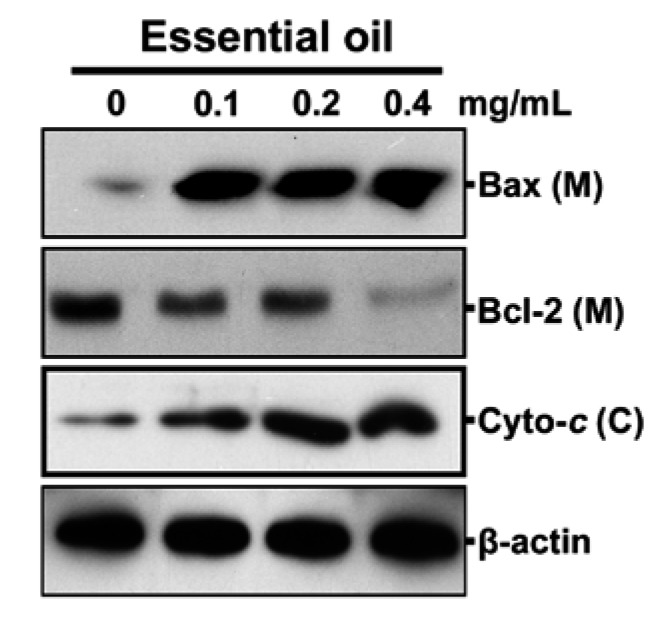
Involvement of mitochondrial stress in the essential oil-mediated apoptosis of KB cells. The cells were treated with the indicated doses of the essential oil for 12 h. Cell lysates were analyzed by 12% sodium dodecyl sulfate-polyacrylamide gel electrophoresis followed by immunoblot analysis. A representative result from three independent experiments is shown. M and C represent the mitochondria and cytosolic fractions, respectively.

Changes in the induction of Bcl-2 family proteins are closely related to an imbalance in the mitochondrial homeostasis, which leads to apoptosis [5,7]. In particular, the release of cytochrome *c *into the cytosol plays an important role in the execution of apoptosis in a number of different cell types, which is tightly regulated by the equilibrium between the antiapoptotic Bcl-2 and pro-apoptotic Bad and Bax [7,18,20]. Essential oil treatment increased the intensity of the bands corresponding to Bax protein in mitochondrial fractions, but significantly reduced the level of Bcl-2 protein (Figure 6). In addition, the amount of cytosolic cytochrome *c *was apparently higher in the cells treated for 12 h with 0.2 mg/mL of the essential oil. Therefore, mitochondrial stress might play a role in the essential oil-mediated apoptosis of KB cells.

## 3. Discussion

Several studies focused on developing effective anticancer and chemopreventive approaches have examined the use of essential oils as natural bioactive substances that can induce sensitive growth inhibition and apoptosis in cancer cells [9,21,22,23,24]. The essential oil of *C. japonica *is a highly purified volatile extract that contains a large number of aromatic components [15,16,17]. Previously, we reported that the essential oil of *C. japonica *is comprised of 68 compounds with the main compounds α-pinene (6.07%), sabinene (8.86%), terpinen-4-ol (9.77%), α-terpineol (6.13%), elemol (11.17%), and 10(15)-cadinen-4-ol (7.16%) demonstrating excellent antimicrobial activity against many types of facultative and obligate anaerobic bacteria [15]. In the present study, we investigated the effect of the essential oil of *C. japonica *on the cell proliferation and induction of apoptosis of KB cells. Apoptosis is a type of physiological cell death that is accompanied by a specialized series of cellular events such as chromatin condensation, DNA fragmentation, cytoplasmic membrane blebbing, and cell shrinkage [10,21,25]. KB cell viability was inhibited by the essential oil in a dose-dependent manner. Also, KB cells treated with the essential oil displayed morphologic changes such as apoptotic bodies and chromatin condensation, as well as DNA fragmentation into oligonucleosomal-sized fragments. In addition, cytometric analysis showed that the cells treated with the essential oil accumulated in the G2/M-phase and S-phase in 12 h, compared to control. Sub-G1 peaks, which represent the cell population containing apoptotic nuclear fragments, were markedly increased after treatment with essential oil in a dose-dependent manner for 12 h.

Apoptosis is a tightly regulated progress that is under the control of several signaling pathways, such as caspase and mitochondrial pathways [2,4,6]. Cytochrome *c* release from mitochondrion to cytosol causes caspase-9-dependent activation of caspase-3 and cleavage of the DNA reparatory protein PARP [20,25,26]. At concentrations more than 0.1 mg/mL, the essential oil induced processing of pro-caspase-3, -8 and -9 and into active forms within 12 h. After caspase-3 activation, some specific substrates for caspase-3 such as PARP are cleaved, which are important for occurrence of apoptosis [26,27]. The present results showed that caspase-3 was activated by cleavage of procaspase during apoptosis of KB cells induced by the essential oil. This suggests that the essential oil-mediated apoptosis of KB cells is caspase-dependent, which was confirmed by the results showing that pretreating the cells with the caspase inhibitors significantly protected the cells from the oil induced cytotoxicity. Furthermore, essential oil also caused specific activation by cleavage of the caspase-3 substrate, PARP, providing further evidence of apoptosis. PARP, a nuclear protein implicated in DNA repair, is one of the earliest proteins targeted for a specific cleavage to the signature 85-kDa fragment during apoptosis [27]. In the apoptotic responses to various cellular stresses, mitochondria play an important role by releasing mitochondrial cytochrome *c *into the cytoplasm [18,20]. Bcl-2 is an integral membrane protein that prevents apoptosis by inhibiting the efflux of the proteins, whereas Bax and Bid are translocated from the cytoplasm to the outer mitochondrial membrane, where they trigger apoptosis via the release of mitochondrial apoptotic factors [28,29]. Therefore, mitochondrial stress mediated by the Bcl-2 family protein is a major event in the execution of apoptotic processes, which is also believed to be closely related to the essential oil-mediated apoptosis in KB cells. This suggests that caspase-independent pathways are also important in the mitochondria-mediated apoptotic mechanism. 

## 4. Experimental

### 4.1. Plant Material and Isolation of the Essential Oil

The aerial parts of *C. japonica* were collected in September, 1999 from the area of Mt. Wansanchilbong in Korea. The plant’s identity was confirmed by Bong-Seop Kil, College of Natural Science, Wonkwang University. A voucher specimen (DJ-99-C20) was deposited at the herbarium of the College of Natural Science, Wonkwang University. The aerial parts of *C. japonica* (1 kg) were air dried and then distilled for 3 h, using a modified Clevenger type apparatus in order to obtain the essential oil. Anhydrous sodium sulphate was used to absorb the little water that the essential oil contained. The essential oil was stored on deep freezer (−70 °C) to minimize the loss of volatile compounds. The essential oil isolated from *C. japonica* [15], was freshly dissolved in the culture dedia.

### 4.2. Cell Culture

KB cells, human oral epidermoid carcinoma cell line (ATCC CCL-17; American Type Culture Collection, Rockville, MD, USA) were grown in Dulbecco’s Modified Eagle medium (DMEM; Gibco, Grand Island, NY, USA) supplemented with 10% heat-inactivated fetal bovine serum (Gibco), 100 U/mL penicillin, and 10 μg/mL streptomycin. KB cells were maintained as monolayer in plastic culture plate at 37 °C in a humidified atmosphere containing 5% CO_2_.

### 4.3. Measurement of Cell Viability

MTT was used to examine the cell viability. Briefly, the cultured KB cells were exposed to the essential oil. At various exposure times, a MTT solution (10 μL, 5 mg/mL in PBS as stock solution) was added to each well, and the cells were incubated for a further 4 h at 37 °C. Seventy microliters of acidic isopropanol was then added to each well, and the absorbance of the plates was read at 560 nm using a Spectra Count TM (Packard Instrument Co., Downers Grove, IL, USA) ELISA reader.

### 4.4. Cell Cycle Analysis

The progression of the cell cycle was determined using flow cytometric analysis after staining with PI. Initially, the suspension (2 × 10^6^ cells) of essential oil treated KB cells was fixed with 80% ethanol at 4 °C for 12 h, and incubated overnight at 4 °C with 1 mL of a PI staining mixture (250 μL of PBS, 250 μL of 1 mg/mL RNase in 1.12% sodium citrate, and 500 μL of 50 μg/mL PI in 1.12% sodium citrate). After staining, 1 × 10^4^ cells were analyzed using the FACS Caliber^®^ system (Becton Dickinson, San Jose, CA, USA).

### 4.5. Nuclear Morphology Analysis

Morphological changes of apoptotic cells were also examined using fluorescence microscopy. At various concentrations after treating KB cells with the essential oil, the cells were harvested, fixed with absolute ethanol, and stained with Hoechst 33258 for 15 min at 37 °C. The cells were then visualized using fluorescence microscopy (Olympus BX50, Tokyo, Japan) with UV excitation at 300-500 nm. Cells containing condensed and/or fragmented nuclei were considered to be apoptotic cells.

### 4.6. DNA Extraction and DNA Gel Electrophoresis

The characteristic ladder pattern of DNA break was analysed by agarose gel electrophoresis. KB cells treated with different concentrations of the essential oil for 12 h were collected, washed with PBS twice, and DNA from KB cells was isolated by a Wizard Genomic DNA purification kit (Promega Co., Madison, WI, USA). Isolated genomic DNA was subjected to 2.0% agarose gel electrophoresis at 100 V for 1 h. DNA was visualized by staining with ethidium bromide under UV light. 

### 4.7. Preparation of Cytosolic and Mitochondrial Extract

The subcellular fractions were prepared as described previously [18]. The harvested pellets were suspended in 100 μL of buffer A [20 mM Hepes-KOH, pH 7.5, 10 mM KCl, 1.5 mM MgCl**_2_**, 1 mM sodium EDTA, 1 mM sodium EGTA, 1 mM dithiothreitol (DTT; Sigma), 1 μg/mL aprotinin, 100 μg/mL phenylmethyl-sulfonylfluoride (PMSF; Sigma), and 250 mM sucrose]. After incubate on ice for 10 min, homogenize cells in an ice-cold dounce tissue grinder (45 strokes) until 70**–**80% of the nuclei did not have the shiny ring and centrifuge at 700 × g for 10 min at 4 °C. The supernatant was collected and further centrifuged at 10,000 × g for 30 min at 4 °C to isolate cytosolin fraction. Cytosolic fraction was stored at −80 °C until ready for Western blotting.

### 4.8. Western Blot Analysis

Equal amount of protein (40 μg/sample) was separated electrophoretically by 10–12% SDS-PAGE and blotted onto PVDF membranes. The blots were probed with the primary antibodies and incubated with a horseradish peroxidase-conjugated anti-IgG in blocking buffer for 1 h. After washing, the blots were developed with ECL (Santa Cruz Biotechnology, Santa Cruz, CA, USA) and exposed to X-ray film (Eastman-Kodak, Rochester, NY, USA). The polyclonal antibodies specific to Bcl-2 (SC-783, 1:200), caspase-3 (SC-7148, 1:200), caspase-8 (SC-6134, 1:100), and PARP (SC-7150, 1:200), and the monoclonal antibodies specific to Bax (SC-7480, 1:100), cytochrome *c *(SC-13156, 1:500), and caspase-9 (SC-17784, 1:100) were purchased from Santa Cruz Biotechnology. The polyclonal antibody specific to β-actin (1:500) was purchased from Sigma Chemical Co. 

### 4.9. Statistical Analysis

All the data are expressed as a mean ± standard error (SE). One-way ANOVA using SPSS ver. 10.0 software was used for multiple comparisons. A *p *< 0.05 was considered significant.

## 5. Conclusions

This study demonstrates that the essential oil of *C. japonica *induces apoptosis through mitochondrial- and caspase-dependent mechanisms. However, identifying the active components responsible for the apoptosis inducing activity is quite difficult because the essential oil of *C. japonica *contains a large number of components [15]. Terpinen-4-ol, α-pinene, α-terpineol, and sabinene are naturally occurring monoterpenes found in the essential oils of many aromatic plants that have been shown to have anticancer effects as well as antioxidant and anti-inflammatory activities [30,31,32,33]. Overall, the essential oil of *C. japonica *may have potential as a cancer chemopreventive and therapeutic agent.
